# Synthesis and Assessment of Two Malonyl Dihydrazide Derivatives as Corrosion Inhibitors for Carbon Steel in Acidic Media: Experimental and Theoretical Studies

**DOI:** 10.3390/molecules26113183

**Published:** 2021-05-26

**Authors:** Saleh S. Alarfaji, Ismat H. Ali, Mutasem Z. Bani-Fwaz, Mahmoud A. Bedair

**Affiliations:** 1 Department of Chemistry, College of Science, King Khalid University, P.O. Box 9004, Abha 61413, Saudi Arabia; ssalarvagi@kku.edu.sa (S.S.A.); mbanifawaz@kku.edu.sa (M.Z.B.-F.); 2 Research Centre for Advanced Materials Science (RCAMS), King Khalid University, P.O. Box 9004, Abha 61413, Saudi Arabia; 3 Department of Chemistry, Faculty of Science (Men’s Campus), Al-Azhar University, Cairo 11884, Egypt; mbedair@ub.edu.sa; 4 College of Science and Arts, University of Bisha, P.O. Box 101, Al-Namas 61977, Saudi Arabia

**Keywords:** corrosion, inhibitors, malonyl dihydrazide, Monte Carlo simulations, Fukui functions

## Abstract

Despite the extensive use of carbon steel in all industrial sectors, particularly in the petroleum industry, its low corrosion resistance is an ongoing problem for these industries. In the current work, two malonyl dihydrazide derivatives, namely 2,2’-malonylbis (*N*-phenylhydrazine-1-carbothiamide (MBC) and *N*’1, *N*’3-bis(-2-hydroxybenzylidene) malonohydrazide (HBM), were examined as inhibitors for the carbon steel corrosion in 1.0 M HCl. Both MBC and HBM were characterised using thin-layer chromatography, elemental analysis, infrared spectroscopy, and nuclear magnetic resonance techniques. The corrosion tests were performed using mass loss measurements, polarisation curves, and electrochemical impedance spectroscopy. It is obtained from the mass loss studies that the optimal concentration for both inhibitors is 2.0 × 10^−5^ mol/L, and the inhibition efficiencies reached up to 90.7% and 84.5% for MBC and HBM, respectively. Electrochemical impedance spectroscopy (EIS) and potentiodynamic polarisation (PDP) indicate an increased impedance in the presence of both MBC and HBM and mixed-type inhibitors, respectively. Both inhibitors can mitigate corrosion in the range of 298–328 K. Values of free energy changes obtained from the Langmuir model suggest that the inhibitors suppress the corrosion process principally by chemisorption. The computational investigations were conducted to identify the factors connected with the anti-corrosive properties of the examined inhibitors.

## 1. Introduction

Acids have various uses in the industrial sector, such as acidification of oil and gas wells, marinating and cleaning metallic products [1,2]. It is well-known that these acids cause severe damage to metals and alloys. Consequently, there is an increasing need for new techniques to suppress corrosion reactions. Chemical inhibitors are among the most robust, inexpensive methods for hindering corrosion reactions [3,4,5,6]. Different organic compounds, such as ionic liquids and pyrazoline derivatives, and most compounds containing heteroatoms are widely exploited as corrosion inhibitors in various industries. These heteroatoms, such as O, S, and N, help the organic compound to be adsorbed quickly and firmly on the metal and alloy surfaces, and hence block the active sites and make a thin layer on the surface, which hinder the path of corrosive media to the surface [7,8,9,10,11]. The adsorption of the organic compound on metals or alloys is controlled by many factors, such as the surface nature, type of the metal charge, and the inhibitor structure. Among organic compounds, hydrazine compounds showed high corrosion inhibition effectiveness [12,13,14,15,16,17,18]. Experimental methods are appropriate for clarifying the inhibition mechanisms and the corrosion behaviour of metals and alloys, but they are time-consuming and expensive. Rapid development in theoretical chemistry and computer software and hardware provides very effective computing and graphical tools for researchers. During the past decade, several corrosive works containing quantum chemical calculations have been published. Such calculations are typically used to explore several properties, such as the electronic properties of the inhibitors, the effect of the energies of the highest and the lowest occupied molecular orbitals (HOMO and LUMO, respectively), and the gap between them [19,20,21]. Theoretical studies including both density functional theory (DFT) calculations and molecular dynamic (MD) simulations on the inhibition action of some hydrazine derivatives have been reported recently [20].

This work aims to explore the corrosion behaviour of C-steel in the presence of two malonyl dihydrazide derivatives, namely, 2,2’-malonylbis(*N*-phenylhydrazine-1-carbothiamide), abbreviated as MBC, and *N*’1, *N*’3-bis(-2-hydroxybenzylidene)malonohydrazide, abbreviated as HBM. It is well-known that the derivatives of Schiff bases are considered as eco-friendly materials [19,20]. In addition, malonic acid is usually used as a green material for various applications [21]. This work also aims to investigate the inhibition effect of these two derivatives and to explain the nature of the inhibition process using the potentiodynamic polarisation curves (PDP), electrochemical impedance spectroscopy (EIS), and scanning electron microscopy (SEM). Additionally, to evaluate the adsorption affinity of the inhibitors under study on the surface of the carbon steel, comprehensive theoretical studies were performed using MD and DFT.

## 2. Results

### 2.1. Characterisation of the Inhibitors 

**Scheme 1:**
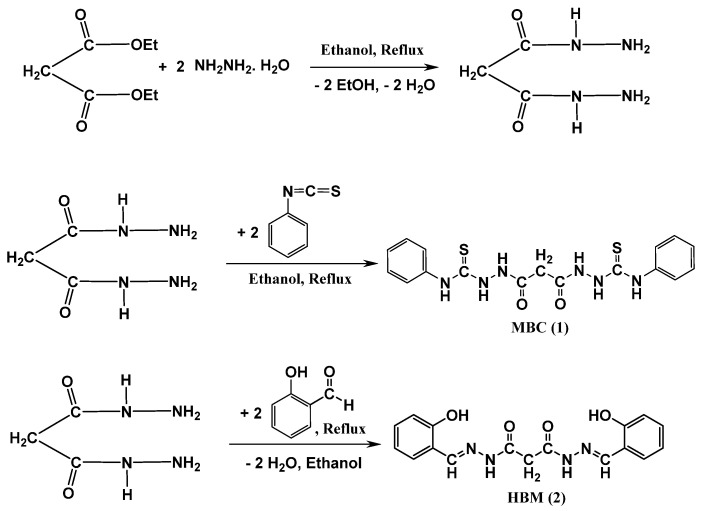
General procedure for the synthesis of both the thiosemicarbazide derivative (1) and hydrazone Schiff base derivative (2).

The synthesis of both the thiosemicarbazide derivative (MBC) and the hydrazone Schiff base derivative (HBM) from malonyl dihydrazide is shown in Scheme 1 according to the published procedures [22,23,24,25]. The purity of both compounds was checked by thin-layer chromatography (TLC) and melting point.

**Scheme 2:**
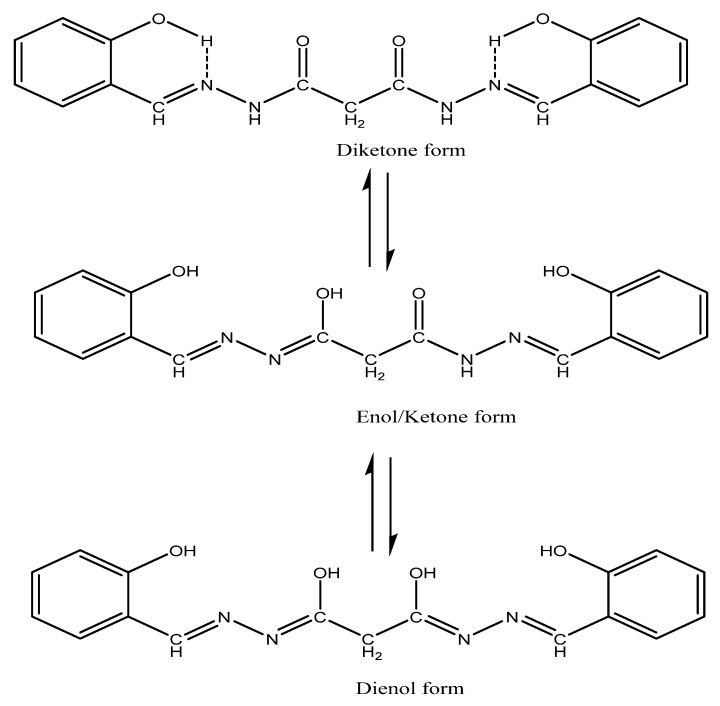
Keto-enol tautomeric isomers of the hydrazone Schiff base.

The number of NMR signals for the hydrazone Schiff base derivative (HBM) can only be explained by assuming at least three different tautomeric isomers, as shown in Scheme 2. Twenty years ago, Zhang et al. [25] confirmed the three different tautomeric isomers by experimental and calculated spectra of NMR spectroscopy.

### 2.2. Weight Loss Measurements

A series of mass loss experiments were performed to determine the corrosion rate in the presence and absence of the two inhibitors under study. The corrosion rate of carbon steel and the MBC and HBM efficiencies as corrosion inhibitors were calculated by the weight loss method in 1.0 M HCl solutions containing various MBC and HBM concentrations at 298 K. The values of the inhibition efficiency, IE_ML_%, were calculated using Equation (1) [26] and are displayed in Table 1.

(1) $${\mathrm{IE}\%}_{\mathrm{ML}}=\frac{{\mathrm{CR}}_{\mathrm{ML}}-{\;\mathrm{CR}}_{\mathrm{ML}}^{o}}{{\mathrm{CR}}_{\mathrm{ML}}}\times 100$$

where ${\mathrm{CR}}_{\mathrm{ML}}$ and ${\mathrm{CR}}_{\mathrm{ML}}^{o}$ are corrosion rates of the carbon steel in the inhibited and uninhibited solutions. Obviously, from the data in Table 1, the inhibition efficiency values are directly proportional to the MBC and HBM concentrations. 

The highest corrosion rate and lowest inhibition efficiency are recorded at low concentrations of both inhibitors, MBC and HBM, and the hazards for the attack of the corrosive media are significantly increased. When the concentrations of MBC and HBM increase by a certain quantity, more protection is observed. The maximum inhibition efficiencies (IE_ML_%) were determined as 90.7% and 84.5% for MBC and HBM, respectively. The inhibitive performance of both MBC and HBM is most likely due to the inhibitors’ adsorption on the carbon steel surface.

### 2.3. Temperature Effect

Numerous mass loss experiments were conducted at the range of 298–328 K in the presence and absence of 2.0 × 10^−5^ mol/L of both inhibitors. Table 2 revealed that the corrosion rate increases for both the blank and the inhibited solutions as the temperature increases. The IE_ML_% drops from 90.7% to 79.4% and from 84.5% to 73.5% for MBC and HBM respectively, as the temperature increases. These results designate that both inhibitors could successfully suppress the corrosion reactions at relatively higher temperatures. Results showed in Table 2 confirm that the mass loss values increase as the temperature increases in both the blank and uninhibited solutions. Table 2 also revealed that IE% values of both inhibitors slightly decreased as the temperature increases. 

**Figure 1:**
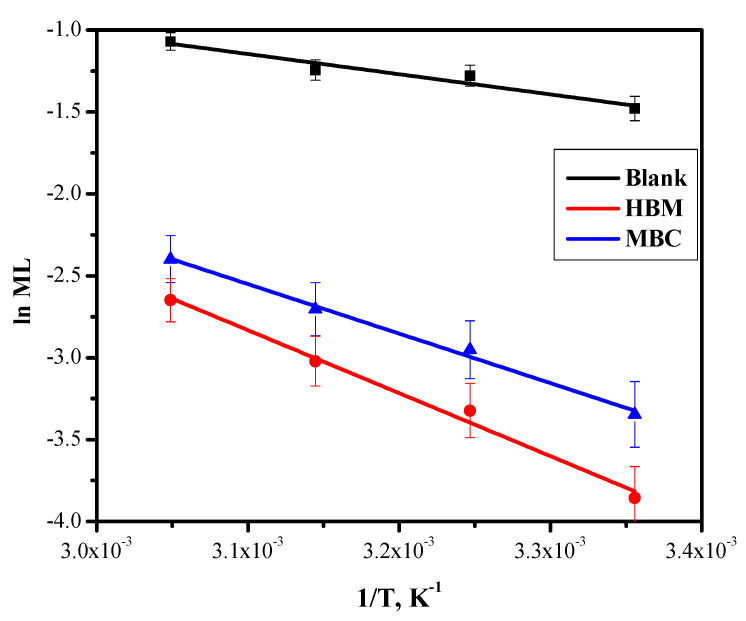
Erying model for blank, MBC and HBM.

The mass loss values were adopted to determine the activation energy values, as shown in Equation (2). The entropy (∆S) and enthalpy (∆H) changes were calculated from the intercept and slope of Equation (3) and Figure 1, respectively.

(2) $$\ln\mathrm{ML}=\ln A\;-\frac{E_{a}}{\mathrm{RT}}$$

(3) $$\ln\frac{\mathrm{ML}}{T}=\frac{-\Delta H}{\mathrm{RT}}+\ln\frac{k_{B}}{h}+\frac{\Delta S}{R}$$

where E_a_ is the activation energy of the corrosion process, k_B_ is the Boltzmann constant, T is the temperature in Kelvin, A is the frequency factor, and h is Planck’s constant.

Values of ΔS, ΔH, and E_a_ in the blank and inhibited solutions are presented in Table 3. The values of activation energies in the presence of both MBC and HBM are lower than that in the blank solution, proving that the corrosion process is more difficult after the addition of the inhibitors. It is also clear that ΔS and ΔH are greater than those in the blank solution. The more significant negative sign of ΔH in the presence of the inhibitors indicates an exothermic nature of the corrosion process, suggesting that the ionisation of steel surface is slow [27] in the presence of both MBC and HBM. A significant and positive value of entropy (ΔS) means that during the rate-determining step, the activated complex is formed via an association rather than a dissociation step [28], confirming that an increase in the disorder takes place when going from reactants to the activated complex.

### 2.4. Adsorption Isotherm Model

Both MBC and HBM are expected to be adsorbed on the carbon steel surface and, hence, protect the metallic surface from the aggressive acidic solution’s attack. Consequently, it is essential to investigate which type of adsorption (physisorption or chemisorption) plays the controlling role during the adsorption process. Generally, inhibitors are adsorbed on metal/solution interface by the displacement of H_2_O molecules, as shown in Equation (4) [3]:

(4) $${\mathrm{inhibitor}}_{\left(\mathrm{solution}\right)}+{\mathrm{xH}}_{2}O_{\left(\mathrm{adsorbed}\right)}\;⇌{\;\mathrm{inhibitor}}_{\left(\mathrm{adsorbed}\right)}+{\mathrm{xH}}_{2}O_{\left(\mathrm{solution}\right)}$$

**Figure 2:**
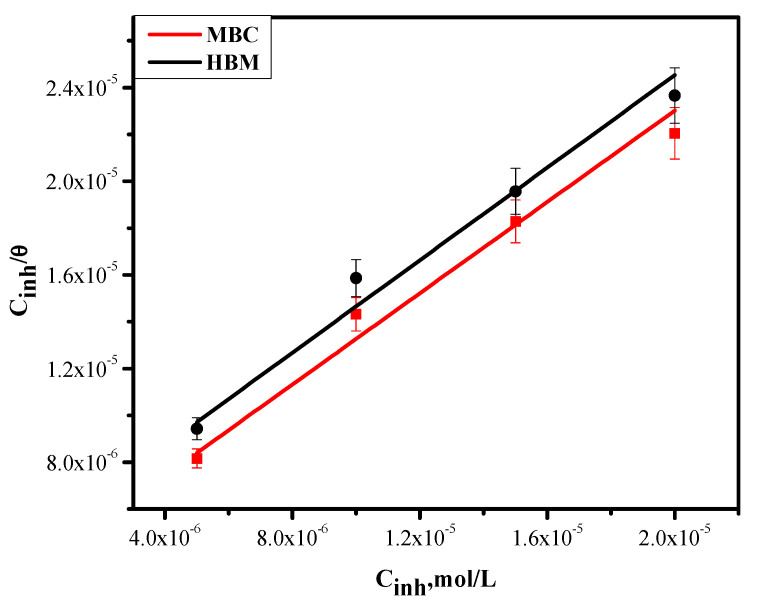
Plot of the Langmuir model for MBC and HBM.

**Figure 3:**
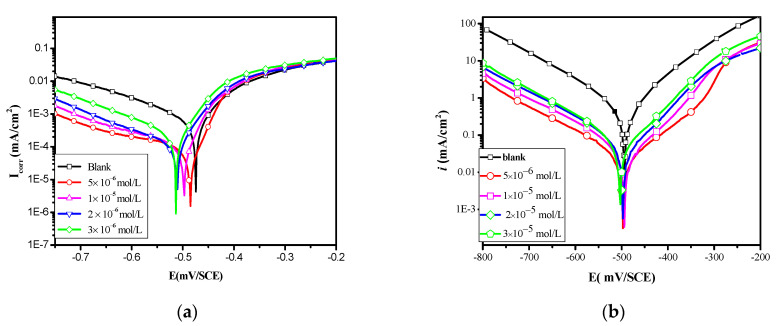
Potentiodynamic polarisation curves of carbon steel in 1.0 M HCl in the presence of different concentrations of (**a**) MBC and (**b**) HBM.

The surface coverage values were found from the mass loss measurement and examined various adsorption models, namely, Langmuir, Temkin, and Freundlich. Results revealed that the Langmuir adsorption model fitted well (Figure 2) with a correlation coefficient (R^2^) of 0.991 and 0.990 for MBC and HBM, respectively. The Langmuir equation is shown in Equation (5) [28]:

(5) $$\frac{C_{\mathrm{inh}}}{Ɵ}=\frac{1}{K_{\mathrm{ads}}}+C_{\mathrm{inh}}$$

where Ɵ is the surface coverage, C_inh_ is the concentration of the MBC and HBM, and K_ads_ is the equilibrium constant of the adsorption. The plot of the Langmuir adsorption model (Figure 3a) gives straight lines having high K_ads_ values, indicating that both MBC and HBM molecules have strong interaction with the steel surface. K_ads_ is directly related to the standard free energy change of adsorption ($\Delta G_{\mathrm{ads}}^{o}$) by Equation (6) [3]:

(6) $$K_{\mathrm{ads}}=\frac{1}{55.5}\mathrm{Exp}\left(\frac{-\Delta G_{\mathrm{ads}}^{o}}{\mathrm{RT}}\right)$$

where 55.5 is the molar concentration of water expressed in mol/L, R is the gas constant, and T is the temperature in Kelvin. The values of K_ads_ and ∆G^o^ are shown in Table 4.

The free energy changes’ negative values designate that adsorption of MBC and HBM on the carbon steel surface is spontaneous. The values of ∆G^o^ are more negative than 40 kJ mol/L, indicating a chemical nature of the adsorption process. The results shown in Table 4 demonstrate that MBC has a slightly higher negative value of ∆G^o^ than that of the HBM inhibitor. This fact can be ascribed to the presence of sulphur atoms in the MBC structure. It actively boosts electron density over the whole molecular skeleton of the MBC inhibitor, and this is one of the potential reasons for the higher efficiency of MBC than that of HBM.

### 2.5. Electrochemical Measurements

#### 2.5.1. Potentiodynamic Polarisation

Potentiodynamic polarisation plots found for carbon steel corrosion in the presence and absence of MBC and HBM are shown in Figure 3a,b The kinetic parameters recorded in Table 5 were obtained from Tafel curves. The percentage of inhibition efficiency (EI%_PDP_) for each inhibitor concentration has been determined by Equation (7):

(7) $$\mathrm{IE}\%=\frac{i_{\mathrm{corr}}-{\;i}_{\mathrm{corr}\left[\mathrm{inh}\right]}}{i_{\mathrm{corr}}}\times 100$$

where i_corr_ and i_corr[inh]_ are the blank and inhibited corrosion current densities, respectively. Table 5 exhibits that in the presence of MBC and HBM, the i_corr[inh]_ is extremely reduced, which indicates the formation of a protective film on the surface of the carbon steel. 

Furthermore, the values of cathodic Tafel slope (b_c_) and anodic Tafel slope (b_a_) presented in Table 5 are almost constant because the hindrance of the corrosion process is principal as a result of the blocking of active positions on the steel surface, and hence, the dissolution mechanism does not change in the presence of inhibitors. 

Inhibitors can be divided into three classes: cathodic, anodic, cathodic, or mixed type according to the displacement of the values of the corrosion potential in the presence and absence of the inhibitor. When the displacement of the corrosion potential’s values is greater than 85 mV, the inhibitor is classified as anodic or cathodic type [3]. The displacement is less than 85 mV for both inhibitors, and thus MBC and HBM are classified as mixed-type inhibitors. Table 5 reveals that the icorr values decrease distinctly as the inhibitor concentration increases. This outcome confirms that both MBC and HBM can act as good corrosion inhibitors. The good inhibition efficiencies for both MBC and HBM are most likely ascribed to their molecular structures, amongst other factors. In Scheme 1, it is observed that both MBC and HBM contain benzene rings, delocalised p-electrons, N atoms, S atoms, and electron-donating groups in their structure, helping the MBC and HBM molecules to adhere firmly to the steel surface. Therefore, high inhibition efficiency is probable.

#### 2.5.2. Electrochemical Impedance Spectroscopy EIS

**Figure 4:**
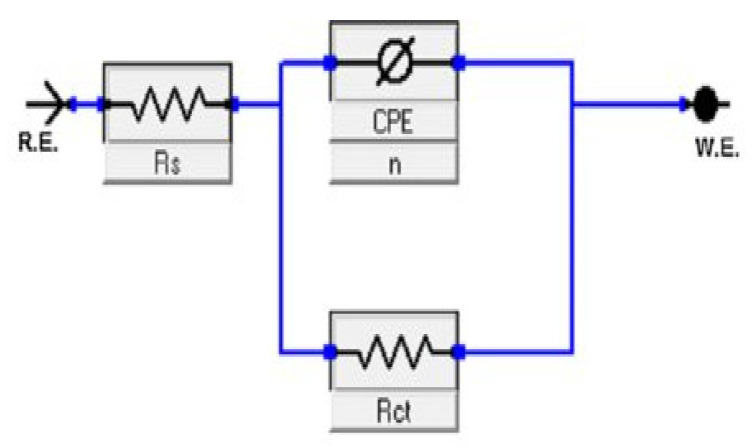
Equivalent circuit model for the analysis of the electrochemical impedance spectroscopy data.

**Figure 5:**
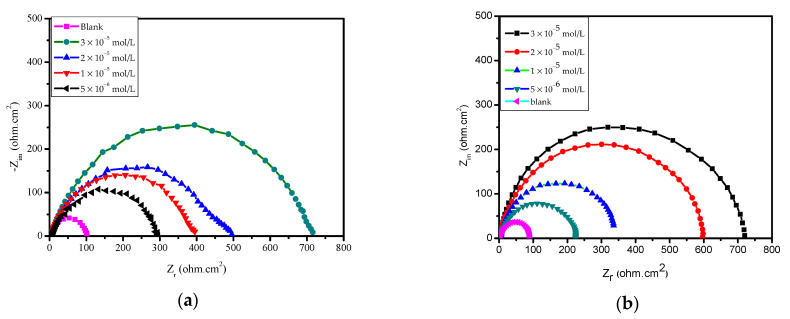
Nyquist spectra for (**a**) MBC and (**b**) HBM.

EIS examined the capacity of MBC and HBM to inhibit the corrosion process. The Electrical Equivalent Circuit (EEC) formula usually used for adjustment is shown in Figure 4. Outcomes are shown in Figure 5a,b. It is clear that the addition of both inhibitors powerfully influenced the impedance response, and this effect is evident from the diameter of semi-circles, which increased clearly.

The Nyquist spectra (Figure 5) exhibit slightly depressed semi-circles at medium to high frequencies and an inductive feature at lower frequencies. The capacitive loop at a higher frequency range suggests that carbon steel’s corrosion is mainly affected by a charge transfer process [29]. The increase of the diameter is more noticeable as the inhibitor concentration increases, which can be ascribed to the formation of a protective film on the steel surface [3]. The inductive loop is ascribed to the relaxation process caused by the adsorption of intermediate products in the presence of acid solutions and inhibitors [3,7]. 

It can be concluded from Table 6 that the R_ct_ values are directly proportional to the inhibitor’s concentrations, and in turn, the values of %IE_EIS_ increase. Values of %IE_EIS_ are obtained from the R_ct_ values of charge transfer resistance data using Equation (8) [29]:

(8) $${\%\mathrm{EI}}_{\mathrm{EIS}}=\frac{R_{\mathrm{ct}}-{\;R}_{\mathrm{ct}\left(\mathrm{inh}\right)}}{R_{\mathrm{ct}}}\times 100$$

where R_ct_ and R_ct(inh)_ are the charge-transfer resistance values without and with inhibitors, respectively.

### 2.6. Quantum Chemical Calculations

#### 2.6.1. Global Reactivity Descriptors

**Figure 6:**
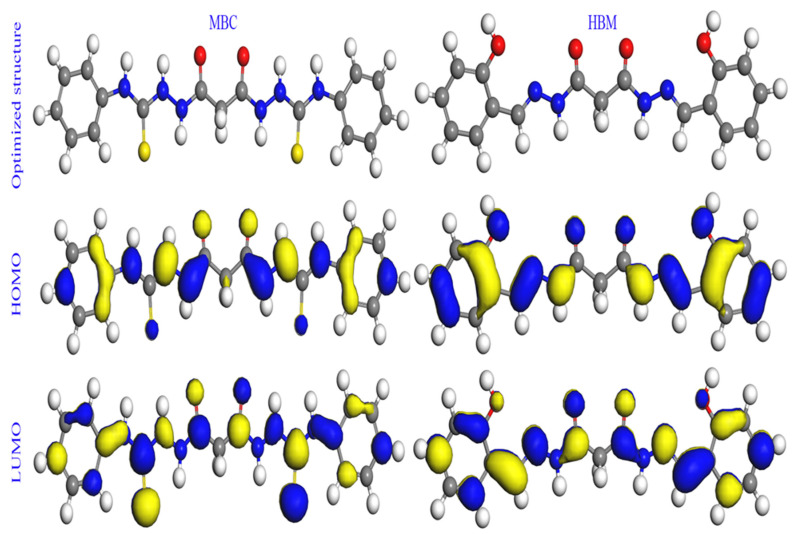
Optimized HOMO and LUMO structures of MBC and HBM obtained from DMol3 calculations.

Quantum chemical calculation can support our findings with highlights on the relation between MBC and HBM molecular configuration and their performance as corrosion inhibitors through various vital data. We found no virtual frequency for the optimised MBC and HBM molecules by examining the calculated data. Figure 6 shows the most stable configuration of MBC and HBM molecules in their neutral conditions, besides the distribution of molecular orbital (LUMO and HOMO) maps. The calculations showed that it varies from 1.38 to 1.40 Å for the two aromatic ring moieties for the bond distance. For the substituted two hydroxyl groups on the aromatic rings in the HBM compound, the O-Ar bond distance was 1.345 Å. The two N-N bond distances were 1.356 Å for MBC and 1.329 Å for the HBM. The two azo methane groups N=C in the HBM compound showed a bond distance of 1.282 Å. The shortest bond distance was observed for the carbonyl groups C=O, with values of 1.229 Å in MBC and 1.207 Å in HBM.

On the other hand, the most extended bond length was observed for C=S groups in MBC (1.665 Å), facilitating its adsorption on the steel surface. The distribution of electron clouds belongs to the HOMO, and LUMO orbitals of the MBC and HBM molecules were located across the entire skeletons. The transfer of the electron is from MBC and HBM to the steel surface, and so protection is mainly a result of the interaction energy between the HOMO—inhibitor and the LUMO—steel, and vice versa. HOMO and LUMO are functions that determine molecules’ capacity for electron-donating and accepting, respectively [30,31,32]. Therefore, we can infer that MBC and HBM can adsorb on the steel surface by multiple active sites, providing significant surface coverage and enhanced protection. 

The different quantum chemical parameters obtained for MBC and HBM are summarised in Table 7. The increase in E_HOMO_ values shows the high electron-donating ability of the molecule to vacant metal orbitals.

On the contrary, the decrease in E_LUMO_ values indicates the high tendency of the molecule to obtain electrons from steel [30,31,32]. The difference between E_LUMO_ and E_HOMO_ is related to the reactivity of the molecule. As the smaller is the value of ΔE, the higher is the molecule’s reactivity [30,31,32]. Regarding our results in Table 7, we can find that MBC possesses more electron-donating tendency (E_HOMO_) than HBM in all forms (neutral and protonated) and phases (gas and aqueous). This tendency to donate electrons supports the order results from the experimental studies. The MBC molecule also possesses the lowest values of E_LUMO_, indicating its high ability to accept electrons from steel (back donation) and therefore strong binding to the steel surface. The E_HOMO_ values of the two molecules increased upon protonation, so the protonated form can be readily adsorbed by donating more electrons. Concerning the energy gap (ΔE), the MBC molecule overcomes HBM in reactivity as it obtains the lowest values. 

The presence of two S atoms and more conjugation in the MBC molecule increases its adsorption ability on the steel surface. The electron back donation from steel to inhibitor molecules was confirmed by negative values of ΔE_Back-donation_ (ΔE_Back-donation_ = $-\frac{\eta }{4}$) [33]. We found that MBC possesses higher total negative charge (−6.755—neutral form—gas phase) than HBM (−5.634) for the electrostatic attraction between the inhibitors and steel surface. This high negative charge on the MBC molecule supports its high inhibition efficiency. Based on the fraction of electrons transferred (ΔN) quantum parameter, positive values of ΔN prove that electrons can follow from inhibitor molecules to the metal surface [34]. ΔN of MBC is higher than HBM, suggest that the tendency of MBC molecules to share their electrons with steel surface is more prominent than HBM molecules. 

#### 2.6.2. Local Reactivity: Fukui Functions

The best way to describe the local sites for electrophilic/nucleophilic reactivity through the atomic charges is the local Fukui indices [35]. It enables us to identify the atomic sites for donating or accepting electrons. Fukui functions are the derivative of chemical potential and can be calculated for nucleophilic and electrophilic attacks by approximation method through the following mathematical relations [36].

(9) $$f\left(r\right)={(\partial \mu /\partial \nu )}_{N}$$

(10) $$f_{k}^{+}=q_{k}\left(N+1\right)-q_{k}\left(N\right)$$

(11) $$f_{k}^{-}=q_{k}\left(N\right)-q_{k}\left(N-1\right)$$

where q_k_ = atomic charge in different states (neutral (N), anionic (N + 1) or cationic (N − 1)). Fukui indices $f_{k}^{+}$ and $f_{k}^{-}$ represent either the nucleophilic or the electrophilic sites of the atoms. The large values of $f_{k}^{-}$ are the most suitable sites to interact with electron-deficient species. On the other hand, the large values of $f_{k}^{+}$ are the most suitable sites to interact with electron-rich species [37]. Table 8 collects the Fukui indices for the MBC and HBM molecules calculated from Hirshfeld atomic charges obtained from the DMol^3^ calculations. As listed in Table 8, the large number of heteroatoms present in MBC molecules (S 10, S 21, N 6, N 17, N 7, N 18, O 2, O 5, N 9, and N 20) obtain high values of $f_{k}^{-}$ (0.034–0.054, aqueous phase). These sites facilitate their interaction with a steel surface. In the HBM molecule, we found less $f_{k}^{-}$ active sites (O 24, N 7, O 25, N 6, N 8, N 9, O 2, and O 5) with lower values (0.036–0.041, aqueous phase). This inefficiency is consistent with that of the experimental ranking MBC > HBM inefficiency. 

The identification of the active site is also confirmed by calculating the dual descriptor, by Equation (12) [38]:

(12) $$\Delta f(k)=f_{k}{}^{+}-f_{k}{}^{-}$$

Positive values of Δ*f* are related to nucleophilic attack but negative values of Δ*f* are related to electrophilic attack. For Δ*f* > 0, the sites highly ready for nucleophilic attack are MBC: C8, C19, S10, and S21, and HBM: N8, N9, C10, and C11. On the contrary, for Δ*f* < 0, the sites highly ready for electrophilic attack are MBC: N6, N7, N17, and N18, and HBM: N6, N7, O24, and O25.

### 2.7. Monte Carlo Simulations

It is imperative to study the interaction between MBC, HBM molecules, and steel surface with Monte Carlo simulations. It was used to determine the different forms of adsorption energy and total energy for MBC and HBM molecules, as presented in Table 9. 

**Figure 7:**
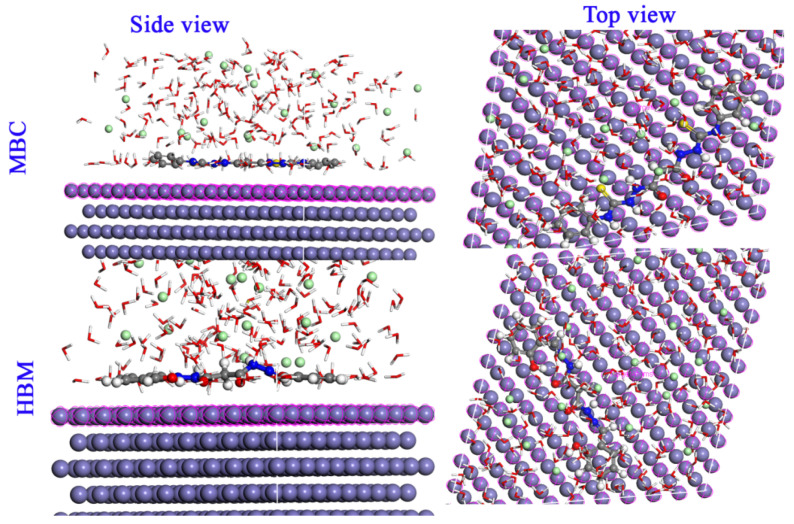
Side and top views of the most appropriate configuration for adsorption of neutral molecules on Fe (110) surface obtained by Monte Carlo simulations in the aqueous solution.

The adsorption energy’s negative values for both MBC and HBM molecules either in neutral or protonated states prove that their adsorption occurred spontaneously [39]. MBC molecules possess higher negative values of adsorption energy (−7285.79 kcal mol^−1^—neutral, −7245.47 kcal mol^−1^—protonated) than HBM molecules (−6473.38 kcal mol^−1^—neutral, −6416.35 kcal mol^−1^—protonated), which suggested the more vital adsorption ability and extra corrosion inhibition of MBC on the Fe (110). The high negative adsorption energy of the protonated forms suggested that MBC and HBM molecules are still highly effective inhibitors even after protonation. Figure 7 shows the equilibrium configuration of MBC and HBM/Fe (110) in the presence of H_2_O, H_3_O^+^, and Cl^−^. It is clear from Figure 7 that simulations of both molecules happened through parallel orientation adsorption on Fe (110) via heteroatoms (O, N, and S), conjugated systems of aromatic moieties, and π electrons. The side-by-side orientation adsorption will provide a higher blocking area and effective interactions between all active sites in MBC and HBM molecules and Fe (110) atoms and stimulate the inhibition effect. The large size of the MBC molecules associated with more adsorption centres, such as the sulphur atoms, resulted in more area than the HBM molecules.

## 3. Materials and Methods

### 3.1. Materials 

The composition of the C-steel used throughout all experiments was (wt%) 0.20 C, 0.045 P, 0.30 Si, 0.53 Mn, 0.055 S, Fe balance. The corrosive solution (1.0 M HCl) was prepared by direct dilution with double-distilled water. 

Malonic diethyl ester, salicylaldehyde, phenyl isocyanate, and hydrazine hydrate (80%) were purchased commercially from Sigma Aldrich, St. Louis MO, USA, and used without any further purification. Absolute ethanol, DMSO, diethyl ether, and all used solvents were purchased from Merck, Mumbai, India. The melting points were reported on a heated stage Gallen Kamp melting point equipment (Gemini Lab, Apeldoom, Netherlands). Elemental C, H, N, and S analyses were carried out on a Fison EA 1108 analyser (Shimadzu, Kyoto, Japan). The infrared (FTIR) spectra were recorded by employing a FTIR.8300 Shimadzu Spectrophotometer (Shimadzu, Kyoto, Japan) by using KBr disc in the frequency range of 4000–400 cm^−1^. The ^1^H NMR spectra were recorded on an Ultra-Shield™ 500 MHz NMR Spectrometer (Shimadzu, Kyoto, Japan) using deuterated DMSO-*d*_6_s the solvent and tetramethylsilane (TMS) as the internal standard. 

### 3.2. Synthesis of Malonyl Dihydrazide

Malonic diethyl ester (38 mL, 40.05 g, 250.06 mmol) in 100 mL absolute ethanol was added slowly dropwise to a stirred excess hot solution of 61 mL of hydrazine hydrate (80%) (1002 mmol) in 100 mL absolute ethanol for 2 h. The reaction mixture was then refluxed in a water bath for 10 h then cooled in an ice-water bath. The white solid was filtrated, rinsed with ethanol and diethyl ether, and then dried in a desiccator over anhydrous CaCl_2_. Yield was 91% (30, 227.07 mmol); mp. 154–155 °C (lit. 154 °C). Elemental analysis: C_3_H_8_N_4_O_2_, Calc.: C 27.27%; H 6.10%; N 42.41%; found: C 27.32%; H 6.06%; N 42.37%. IR (KBr; cm^−1^): 3280, 3188 ν(NH_2_), 3064 ν(NH), 2967 ν(CH), 1671 ν(C=O). ^1^H NMR [(DMSO-*d*_6_, δ)]: δ 2.90 (2H, s, CH_2_), δ 4.24 (4H, s, 2NH_2_), δ 9.07 (2H, s, 2NH) [22].

### 3.3. Synthesis of 2,2’-Malonylbis(N-phenylhydrazine-1-carbothioamide) (MBC)

Phenyl isothiocyanate (2 mL, 2.26 g, 16.75 mmol) in 25 mL absolute ethanol was added slowly dropwise to a stirred hot suspension of malonyl dihydrazide (1.10 g, 8.33 mmol) in 25 mL absolute ethanol. The reaction mixture was then refluxed in a water bath for 8 h then cooled in an ice-water bath. The white solid was assembled and rinsed with ethanol prior to drying in an electric oven at 70 °C. Yield was 92.5% (3.1 g, 7.70 mmol); mp. 181–182 °C (lit. 181–182 °C). Elemental analysis: C_17_H_18_N_6_O_2_S_2_, Calc.: C 50.73%; H 4.51%; N 20.88%; found: C 50.80%; H 4.47%; N 20.90%. IR (KBr; cm^−^^1^): 3144 ν(NH), 3019 ν(ArCH), 1671 ν(C=O), 1349 ν(C=S), 1511 (ArC=C). ^1^H NMR [(DMSO-*d*_6_, δ)]: δ 3.33 (2H, s, CH_2_), δ 7.17–7.50 (10H, m, ArH)), δ 9.64 (2H,s, 2NH), δ 9.84 (2H, s, 2NH), δ 10.36 (2H, s, 2NH) [23].

### 3.4. Synthesis of N’1, N’3-bis(2-Hydroxybenzylidene)malonohydrazide (HBM)

Thin drops of glacial acetic acid were added to a solution of salicylaldehyde (3.5 mL, 4.07 g, 33.36 mmol) in 25 mL absolute ethanol (soln. A). A suspension of malonyl dihydrazide (2.104 g, 15.92 mmol) was added to 25 mL of absolute ethanol (soln. B). Solution A was added to a hot solution B with stirring over 1 h. The reaction mixture was then refluxed in a water bath for 8 h then cooled in an ice-water bath. The white solid was obtained and soaked with ethanol prior to drying in an electric oven at 70 °C. Yield was 88.6% (4.8 g, 14.10 mmol); mp. 233–235 °C (lit. 234–236 °C). Elemental analysis: C_17_H_16_N_4_O_4_, Calc.: C 60.0%; H 4.74%; N 16.46%; found: C 60.05%; H 4.69%; N 16.39%. IR (KBr; cm^−^^1^): 3748; 3271; 3062 ν(NH) + ν(OH), 2963 ν(CH), 1667 ν(C=O), 1607; 1541 ν(C=N) + Amide(**II**). ^1^H NMR (DMSO-*d*_6_, δ): δ 3.36, 3.61, 3.91 (2H, s, CH_2_), ratio 1:2:1, three tautomers, diketone: enol-ketone: dienol forms, δ 6.69–6.74, δ 6.85–6.93, δ 7.17–7.30, δ 7.54–7.66 (8H, 4 sets of multiplets, ArH), δ 8.27, 8.28, 8.42 (2H, s, HC=N-NH, HC=N-NH/HC=N-N=C, HC=N-N=C) ratio 1:1:1, δ 9.01, 10.01 (2H, s), δ 11.07, 11.41 (2H, s), δ 11.42, 11.49 (2H, s) and 11.88 (2H, s) (Ar-OH, -C-OH, =N-NH-C) [24,25].

### 3.5. Electrochemical Measurements 

Electrochemical tests were performed using a three-electrode thermo-stated cell assembly using a Gamry potentiostat/galvanostat/ZRA (model Interface 1000, Gamry, Warminster, PA, USA). A saturated calomel electrode (SCE) and platinum were used as reference and counter electrodes, respectively. The working electrode was manufactured from carbon steel. The electrodes were abraded with various emery papers, degreased with acetone, rinsed with double-distilled water, and dried. All experiments were performed at 25 ± 0.1 °C. The potentiodynamic curves were recorded from −300 to +300 mV at a scan rate of 1 mV S^−1^. The open-circuit potential (OCP) was obtained after 45 min. The electrochemical impedance spectroscopy (EIS) test was carried out using the same instrument. Gamry framework software (version 6.0, Gamry, Warminster, Pennsylvania, USA) was used for conducting the experiments. Echem Analyst Software (Gamry, Warminster, Pennsylvania, USA) was used for plotting and fitting data. EIS measurements were carried out in a frequency range of 10 MHz to 100 kHz with an amplitude of 5 mV peak-to-peak using A.C. signals at relevant corrosion potential.

### 3.6. Computational Study

Supporting the experimental results with the computational calculations is quite helpful to relate the inhibitory effect of the investigated compounds with their geometrical and chemical structures. With the aids of quantum calculations, we can investigate the active sites responsible for the corrosion inhibition mechanism. We reported a complete geometry optimisation for MBC and HBM compounds through the ADF2020.101 and the DMol3 module as a part of the Materials Studio software version of 2017 (Dassault Systems Materials studio, San Diego, CA, USA). The initial optimisation and vibrational frequencies were calculated using the ADF software (Software for Chemistry & Materials, Amsterdam, Netherlands). Further investigations were carried out using the DMol3. The parameters of the DMol3 module during calculation were set as Task = Geometry Optimisation, Functional = LDA and PWC, Basis = DND (4.4), and COSMO control for solvent considerations. For the neutral and protonated form of MBC and HBM compounds, E_HOMO_ and E_LUMO_ as basic potent descriptors as well as some other parameters derived from them were excluded according to the following mathematical relations:

Ionization potential, I = −E_HOMO_ electron affinity, A = −E_LUMO_ (13)

(14) $$\mathrm{electro}\;\mathrm{negativity},\;Χ=-\;\mathrm{chemical}\;\mathrm{potential}\;\left(\mu \right)=\frac{\left(I+A\;\right)}{2}$$

(15) $$\mathrm{absolute}\;\mathrm{hardness},\;\eta =\frac{\left(\;I-A\right)}{2}\;\mathrm{separation}\;\mathrm{energy},\;\Delta E=E_{\mathrm{LUMO}}-E_{\mathrm{HOMO}}$$

(16) $$\mathrm{electrophilicity}\;\mathrm{index},\;\omega =\frac{\mu^{2}}{2\eta }\;\mathrm{nucleophilicity}\;\mathrm{index},\;\varepsilon =\frac{1}{\omega }$$

(17) $$\mathrm{fraction}\;\mathrm{of}\;\mathrm{electrons}\;\mathrm{transferred},\;\Delta N=\;[f-\chi_{\mathrm{inh}}{]/[2(\eta }_{\mathrm{Fe}}{+\eta }_{\mathrm{inh}})]$$

After the optimisation process, the adsorption of MBC and HBM molecules on the Fe (110) surface was simulated using the adsorption locator module. The initial step is constructing a 10 × 10 × 10 3D Fe (110) model. MBC and HBM molecules were allowed to simulate on Fe (110) surface beside other molecules to get a close view of the experimental results, such as H_2_O (200 molecules), H_3_O^+^ (20 molecules), and Cl^−^ (20 molecules). The parameters of Monte Carlo simulations were set as: Task = Simulated annealing, Force-field = COMPASS, Electrostatic = Ewald, and Van Der Waals = Atom-based.

## 4. Conclusions

This work aimed to assess two malonyl dihydrazide derivatives’ efficiency as corrosion inhibitors for carbon steel in acidic media. The two inhibitors were studied experimentally and theoretically for carbon steel corrosion. It is conceivable to conclude the following facts:

❖ MBC and HBM are suitable inhibitors for carbon steel corrosion in acidic media.
❖ The PDP curves show that both MBC and HBM are mixed inhibitors.
❖ EIS investigations proved that the addition of MBC and HBM to the corrosion medium increases the inhibition efficacy.
❖ The study of the impact of temperature on the inhibition efficiency displays that it increases with the temperature increase.
❖ This study revealed that the adsorption of both MBC and HBM molecules on the carbon steel surface has a chemical nature.
❖ The theoretical studies for both MBC and HBM have further supported the experimental results.

## Figures and Tables

**Table 1:** Mass loss results of MBC and HBM inhibitors at 298 K.

Inhibitor	10^6^ Concentration mol/L	Weight Loss (mg)	IE_ML_ (%)	Surface Coverage (θ)
Blank	--	67.9	-	-
MBC	5.0	26.3	61.3	0.6127
	10.0	20.5	69.8	0.6981
	15.0	12.2	82.0	0.8203
	20.0	6.3	90.7	0.9072
HBM	5.0	31.9	53.0	0.5301
	10.0	25.1	63.0	0.6303
	15.0	16.0	76.4	0.7664
	20.0	10.5	84.5	0.8453

**Table 2:** Effect of temperature on the inhibition efficiency of MBC and HBM at 2 × 10^−5^ M.

Inhibitor	Temperature	Weight Loss (mg)	IE (η%)
Blank	298	67.9	-
	308	85.7	-
	318	91.6	-
	328	112.5	-
MBC	298	6.3	90.7
	308	11.1	87.0
	318	15.5	83.1
	328	23.2	79.4
HBM	298	10.5	84.5
	308	16.1	81.2
	318	21.3	76.7
	328	29.8	73.5

**Table 3:** Values of activation parameters for carbon steel in 1.0 M HCl in the absence and presence of 2.0 × 10^−5^ M of MBC and HBM.

Inhibitor	E_a_ (kJ/mol)	∆H (kJ/mol)	∆S (J/mol)
Blank	12.9	−10.3	175
HBM	27.7	−25.1	144
MBC	34.6	−40.3	122

**Table 4:** Values of $K_{ads}$ and $\Delta G_{ads}$.

Inhibitor	$K_{ads}$	$\Delta G_{ads}^{o}\;(\mathrm{kJ}/\mathrm{mol})$
HBM	1.7 × 10^5^	−45.1
MBC	2.5 × 10^5^	−47.9

**Table 5:** Kinetic data obtained from the PDP curves for blank, MBC, and HBM.

Inhibitor	Concentration mol/L	−E_corr_ (mV vs. SCE)	i_corr_ (μA cm^−2^)	β_a_ (mV dec^−1^)	β_c_ (mV dec^−1^)	θ	IE%
Blank	---	335	327	62	124		
MBC	5.0	378	129	81	126	0.6055	60.6
	10	367	88	67	131	0.7309	73.1
	20.0	383	58	101	157	0.8226	82.3
	30.0	371	32	92	163	0.9021	90.2
MBC	5.0	367	147	117	182	0.5504	55.0
	10.0	379	97	92	127	0.7034	70.3
	20.0	356	71	88	136	0.7828	78.3
	30.0	341	49	82	125	0.8502	85.0

**Table 6:** Results obtained from EIS diagram for MBC and HBM.

Inhibitors	10^6^ × Conc. (mol/L)	R_S_, (Ω cm^2^)	10^−3^ × Y_ο_ μ Ω^−1^ sn cm^−1^	n	R_ct_ (Ω cm^2^)	Θ	%IE_EIS_
Blank	--	11.9	134.10	0.799	19.3 ± 0.7	-	-
MBC	5.0	9.5	123.40	0.801	49.4 ± 1.7	0.6093	60.9
	10.0	9.6	148.70	0.827	72.5 ± 3.1	0.7337	73.4
	20.0	1.5	33.22	0.866	111.8 ± 4.5	0.8274	82.7
	30.0	1.7	33.01	0.835	219.4 ± 7.8	0.9120	91.2
HBM	5.0	2.7	85.72	0.887	43.1 ± 1.8	0.5522	55.2
	10.0	10.0	90.12	0.750	64.4 ± 2.6	0.7003	70.0
	20.0	9.9	163.6	0.791	82.7 ± 3.7	0.7666	76.7
	30.0	1.4	39.83	0.862	140.7 ± 6.3	0.8628	86.3

R_s_ is the solution resistance, R_ct_ is charge-transfer resistance, Y_ο_ is the capacitance, and n is the phase shift that can be explained as the degree of the surface inhomogeneity [20].

**Table 7:** The calculated quantum chemical parameters in eV for the neutral and protonated inhibitors at DMol3 in gas phase and in aqueous phase.

Molecule		E_HOMO_	E_LUMO_	∆E	∆E_back donation_	T.E.	ε	σ	ω	χ	∆N	∆N_max_	η	IE_EIS_
		(eV)	(eV)	(eV)	(eV)	(eV)	(eV^−1^)	(eV^−1^)	(eV)	(eV)	(e)	(e)	(eV)	(%)
Neutral														
gas phase	MBC	−4.8178	−1.8514	2.9664	−0.3708	−52,259.80	0.2668	0.6742	3.7484	3.3346	1.2356	1.1241	1.4832	91.2
	HBM	−5.0035	−1.6499	3.3536	−0.4192	−31,753.34	0.3030	0.5964	3.3000	3.3267	1.0953	0.9920	1.6768	86.3
aqueous	MBC	−5.3499	−2.3952	2.9547	−0.3693	−52,261.09	0.1970	0.6769	5.0754	3.8725	1.0585	1.3106	1.4774	91.2
	HBM	−5.2743	−1.9849	3.2894	−0.4112	−31,755.13	0.2497	0.6080	4.0049	3.6296	1.0246	1.1034	1.6447	86.3
Protonated														
gas phase	MBC-H^2+^	−10.5274	−9.4107	1.1167	−0.1396	−52,266.230	0.0112	1.7910	88.997	9.9691	−2.6588	8.9273	0.5583	91.2
	HBM-H^2+^	−10.7963	−8.1222	2.6741	−0.3343	−31,769.026	0.0299	0.7479	33.460	9.4593	−0.9196	3.5373	1.3371	86.3
aqueous	MBC-H^2+^	−5.9557	−4.1612	1.7945	−0.2243	−52,271.259	0.0701	1.1145	14.258	5.0584	1.0819	2.8188	0.8973	91.2
	HBM-H^2+^	−6.1254	−3.5374	2.5879	−0.3235	−31,776.083	0.1109	0.7728	9.0196	4.8314	0.8380	1.8669	1.2940	86.3

**Table 8:** The Fukui functions of studied inhibitors calculated by the DMol3 method.

Atom	MBC						Atom	HBM					
	Gas Phase			Aqueous Phase				Gas Phase			Aqueous Phase		
	$f^{+}$	$f^{-}$	Δf	$f^{+}$	$f^{-}$	Δf		$f^{+}$	$f^{-}$	Δf	$f^{+}$	$f^{-}$	Δf
C (1)	0.028	0.018	0.01	0.033	0.024	0.009	C (1)	0.025	0.015	0.01	0.032	0.019	0.013
O (2)	0.035	0.041	−0.006	0.034	0.041	−0.007	O (2)	0.039	0.037	0.002	0.04	0.036	0.004
C (3)	0.01	0.008	0.002	0.012	0.012	0	C (3)	0.009	0.007	0.002	0.012	0.009	0.003
C (4)	0.028	0.018	0.01	0.033	0.024	0.009	C (4)	0.025	0.015	0.01	0.032	0.019	0.013
O (5)	0.035	0.041	−0.006	0.034	0.041	−0.007	O (5)	0.039	0.037	0.002	0.04	0.036	0.004
N (6)	0.02	0.042	−0.022	0.026	0.05	−0.024	N (6)	0.014	0.038	−0.024	0.019	0.039	−0.02
N (7)	0.011	0.041	−0.03	0.015	0.046	−0.031	N (7)	0.014	0.038	−0.024	0.019	0.04	−0.021
C (8)	0.046	0.01	0.036	0.051	0.016	0.035	N (8)	0.044	0.029	0.015	0.053	0.039	0.014
N (9)	0.017	0.029	−0.012	0.021	0.034	−0.013	N (9)	0.044	0.029	0.015	0.053	0.039	0.014
S (10)	0.095	0.063	0.032	0.097	0.054	0.043	C (10)	0.049	0.032	0.017	0.057	0.032	0.025
C (11)	0.012	0.012	0	0.018	0.017	0.001	C (11)	0.049	0.032	0.017	0.058	0.032	0.026
C (12)	0.017	0.019	−0.002	0.021	0.021	0	C (12)	0.017	0.024	−0.007	0.022	0.031	−0.009
C (13)	0.02	0.019	0.001	0.016	0.015	0.001	C (13)	0.017	0.024	−0.007	0.023	0.032	−0.009
C (14)	0.037	0.037	0	0.029	0.03	−0.001	C (14)	0.02	0.027	−0.007	0.024	0.032	−0.008
C (15)	0.023	0.022	0.001	0.018	0.016	0.002	C (15)	0.026	0.023	0.003	0.022	0.022	0
C (16)	0.019	0.02	−0.001	0.019	0.021	−0.002	C (16)	0.048	0.04	0.008	0.041	0.034	0.007
N (17)	0.02	0.042	−0.022	0.026	0.05	−0.024	C (17)	0.026	0.037	−0.011	0.021	0.036	−0.015
N (18)	0.011	0.041	−0.03	0.015	0.046	−0.031	C (18)	0.029	0.021	0.008	0.03	0.02	0.01
C (19)	0.046	0.01	0.036	0.051	0.016	0.035	C (19)	0.029	0.02	0.009	0.03	0.021	0.009
N (20)	0.017	0.029	−0.012	0.021	0.034	−0.013	C (20)	0.026	0.037	−0.011	0.022	0.036	−0.014
S (21)	0.095	0.063	0.032	0.097	0.054	0.043	C (21)	0.048	0.04	0.008	0.041	0.034	0.007
C (22)	0.012	0.012	0	0.018	0.017	0.001	C (22)	0.026	0.023	0.003	0.022	0.022	0
C (23)	0.019	0.02	−0.001	0.019	0.021	−0.002	C (23)	0.02	0.027	−0.007	0.024	0.033	−0.009
C (24)	0.023	0.022	0.001	0.018	0.016	0.002	O (24)	0.016	0.034	−0.018	0.019	0.041	−0.022
C (25)	0.037	0.037	0	0.029	0.03	−0.001	O (25)	0.016	0.034	−0.018	0.018	0.04	−0.022
C (26)	0.02	0.019	0.001	0.016	0.015	0.001							
C (27)	0.017	0.019	−0.002	0.021	0.021	0							

**Table 9:** The outputs and descriptors calculated by the Monte Carlo simulation for adsorption of MBC and HBM on Fe (110) (in kcal/mol).

Inhibitor	Neutral		Protonated	
	MBC	HBM	MBC-H^2+^	HBM-H^2+^
Total energy (kcal mol^−1^)	−6316.14	−6315.12	−6181.61	−6164.68
Adsorption energy (kcal mol^−1^)	−7285.79	−6473.38	−7245.47	−6416.35
Rigid adsorption energy (kcal mol^−1^)	−6358.46	−6345.46	−6318.53	−6228.34
Deformation energy (kcal mol^−1^)	−927.33	−127.91	−926.94	−188.01
(dE_ads__/__dNi_) (kcal mol^−1^)	−1197.17	−379.55	−1378.37	−629.96
Binding energy (kcal mol^−1^)	7285.79	6473.38	7245.47	6416.35
IE_EIS_ (%)	91.2	86.3	91.2	86.3

## Data Availability

Data are available from the authors upon request.

## Sample Availability

Samples of the compounds 2,2’-malonylbis(*N*-phenylhydrazine-1-carbothiamide) and *N*’1, *N*’3-bis(-2-hydroxybenzylidene)malonohydrazide are available from the authors.
